# Clinically silent indolent T‐cell leukemia

**DOI:** 10.1002/ccr3.1528

**Published:** 2018-11-10

**Authors:** Clément Janot, Joffrey Feriel, Claire Borie, Edouard Lefevre, Annelise Bennaceur‐Griscelli, Ali G. Turhan, Cédric Aumont

**Affiliations:** ^1^ Division of Hematology Centre Hospitalier Universitaire Université Paris Sud 11 Le Kremlin Bicêtre France; ^2^ INSERM U935, Campus CNRS Villejuif France; ^3^ Department of Internal Medecine Centre Hospitalier Universitaire Université Paris Sud 11 Le Kremlin Bicêtre France

**Keywords:** Cellular hematology, indolent, lymphoid neoplasms, T‐cell leukemia, T‐cell prolymphocytic leukemia

## Abstract

The clinically silent, symptom‐free T‐cell prolymphocytic leukemia case that we report here confirms the major interest of the analysis of the blood smear as usual care of any emergent lymphocytosis. It also brings out the issue of the monitoring and follow‐up of this uncommon presentation.

## Background

T‐cell prolymphocytic leukemia (T‐PLL) is a rare and severe mature lymphoid malignancy characterized by a clonal proliferation of a mature T‐cell population, classically associated with a serious vital prognosis. The clinical presentation usually includes hepatosplenomegaly, associated with peripheral adenopathy, asthenia, but also skin lesions and peritoneal or pleural effusion in some advanced cases. The disease occurs essentially in elderly patients with an average age of 70 at time of diagnosis, and the median survival is estimated to be <1 year. Indeed, T‐PLL is usually resistant to classic antineoplastic therapies.

Blood counts typically reveal a progressive lymphocytosis with the typical morphology consisting of prolymphocytes of small to medium size with oval or markedly irregular nuclei, condensed nuclear chromatin, a single prominent nucleolus, and intensely basophilic nongranular cytoplasm with cytoplasmic protrusions or “blebs.” In 25% of cases, the cell size is smaller and the nucleolus may not be visible by light microscopy (small cell variant). In 5%, the nuclear outline is markedly irregular and can even be cerebriform, mimicking Sezary cells, or can remind a “flower cell” aspect as well in 5% of cases.

The phenotypic profile of these clonal T cells has been described: leukemic T cells are CD2+, CD3+, CD4+, CD5+, CD7+ with a strong expression of CD7, CD52, and TCR *α* or *β* chains. 60% of the clones are CD4+ CD8−, 25% coexpress CD4 and CD8, and only 15% are CD4− CD8+ 1 Various chromosomal abnormalities generally involving chromosome 14 (but also chromosomes 11, 8) are usually identified 2.

Here, we report the unusual case of a 75‐year‐old patient in whom a T‐PLL was discovered during the biological exploration of a chronic and particularly clinically silent lymphocytosis, fortuitously revealed by a routine checkup.

## Case Presentation

A 75‐year‐old patient was referred to internal medicine consultation in June 2016 for exploration of a stable moderate lymphocytosis, first documented in 2014 with 7.17 G/L. Two years later, lymphocyte counts rose to 10.1 G/L. Past history included diabetes, a right partial nephrectomy for malformation, a heavy smoking of 40 years generating leukoplakia on the vocal cords, and high blood pressure. His treatment included insulin, gliclazide, perindopril, bisoprolol, nicardipine, indapamide, urapidil, pravastatin, aspirin, and oral potassium. Blood analysis found a slightly increased uric acid (453 *μ*mol/L), as well as calcium (2.66 mmol/L). Renal function as well as liver function tests were normal. Serum protein electrophoresis was normal but immunofixation showed a low‐key oligoclonal profile of IgG and IgM.

Complete blood counts showed hemoglobin 14.0 g/dL, MCV 99 fL, MCHC 32.6 g/dL, platelet count at 200,000/mm^3^, WBC at 15,630/mm^3^ with neutrophils at 4890/mm^3^, lymphocytes at 9070/mm^3^, and monocytes at 1400/mm^3^.

**Figure 1:**
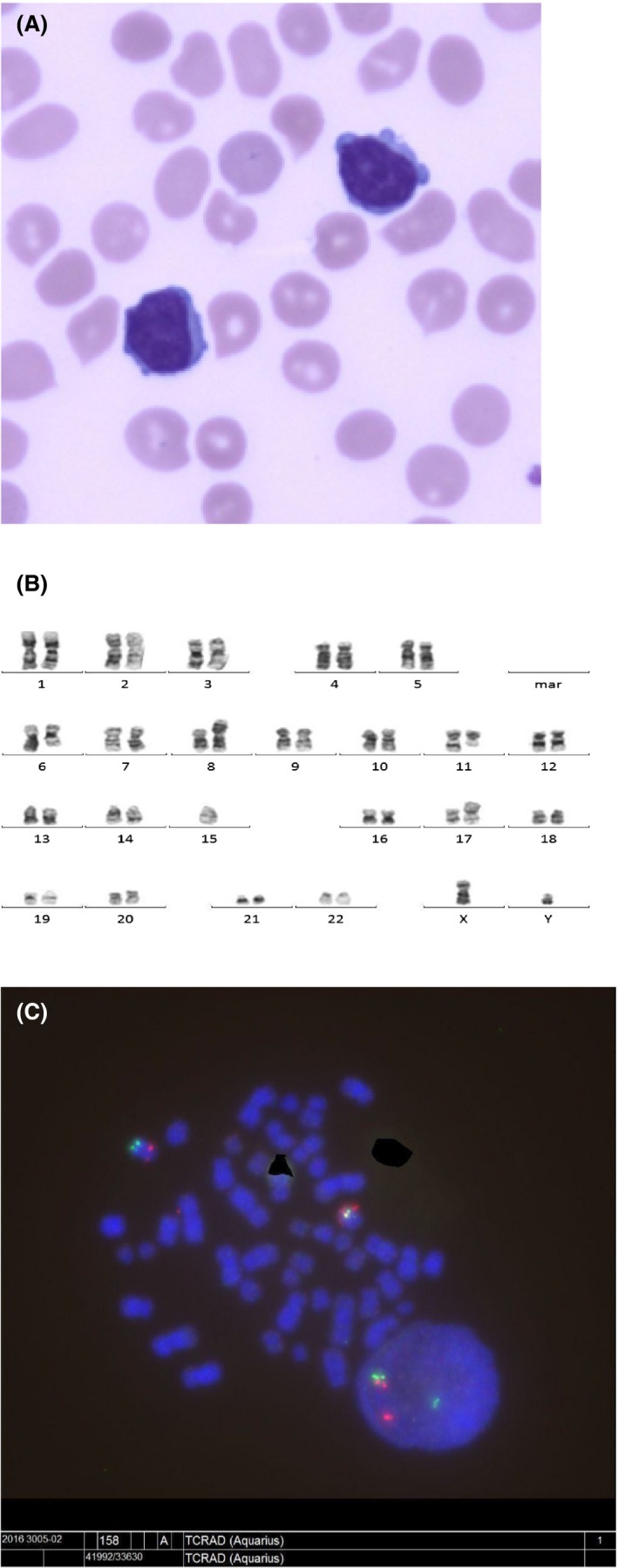
(A) A microphotograph of the blood smear showing small lymphoid cells with dense chromatin. Their nucleoli and their cytoplasmic expansions suggested the hypothesis of a T‐prolymphocytic neoplasm. (B) Complex karyotype involving no less than six pairs of chromosomes, forming a typical profile of T‐PLL. (C) FISH analysis.

The analysis of blood smear showed a monomorphic lymphocytic population composed of small mature cells, with high nuclear/cytoplasmic ratio, with mature chromatin and prominent nucleoli, presenting regularly basophilic expansion of the cytoplasm. Cytoplasmic expansions (blebs) were present (Fig. 1A). Peripheral lymphoid immunophenotyping demonstrated a clear CD4+/CD8+ imbalance with a ratio of 16, caused by clonal population of CD3+/CD4+ representing 80% of lymphocytes. Antigenic profile revealed a naive T‐cell population that widely expressed CD45RA and CCR7 (92%), T‐markers such as CD2, CD3, CD5, and CD7, but did not express CD8, CD16, CD25, CD56, or CD57. The proportion of TCR‐V*β*21‐3 variant was increased at 88% (reference range 1.53–4.70%), whereas TCR‐*β* of CD8+ cells and TCR‐*α* relative proportions were normal. NK‐cells and B‐cells populations were qualitatively normal.

The diagnosis of a lymphoid neoplasm and a T‐prolymphocytic leukemia (T‐PLL) was suspected. On clinical examination, the patient was in good general health with stable body weight at 100 kg. His temperature was of 37°C, and blood pressure was 154/84 mmHg. TEP scan confirmed the lack of hepatosplenomegaly.

The marrow aspirate revealed the presence of 40% of atypical lymphocytes of small size, with high nuclear/cytoplasmic ratio, uniform mature chromatin with a single nucleolus in most of them. Bone marrow karyotype showed a complex karyotype with inversion on chromosome 14 (impacting on locus 14q11) and deletion on the chromosome 11 long arm (impacting on locus 11q23, id ATM), isochromosomes 6p and 8q, causing partial trisomy and monosomy, respectively on its long and short arm. Furthermore, these abnormal nuclei could be subdivided in two different type, as a first one carried an additional monosomy of chromosome 15, and a second carried a rearrangement in both long arms of chromosomes 1 and 3, suggesting a potential translocation t(1;3) (Fig. 1B). FISH analysis with TCRAD break apart probe confirmed inversion on chromosome 14 in 35% of nuclei, deletion of ATM in 35% of nuclei too and any deletion of the TP53 gene. Among these abnormalities, 14 inversion is a characteristic lesion of T ‐PLL, which is frequently included in complex karyotype. ATM involvement is also recurrent 3 (Fig. 1C).

Overall, the biological abnormalities strongly suggested the diagnosis of T‐PLL despite the excellent clinical setting and absence of any lymphadenopathy or splenomegaly. No chemotherapy was initiated, and the patient is in clinical follow‐up in an outpatient setting.

## Discussion

T‐cell PLL is an usually aggressive disease presenting with high mortality rate 4. The patient described in this report had an unusual presentation with no symptoms with unexpected discovery of a T‐prolymphocytic clone. The patient appeared strictly asymptomatic at the time of diagnosis; he did not show any deep or peripheral organomegaly and was apyretic, and his overall condition was maintained. The model of a biphasic chronology was previously suggested for this pathology, containing a relatively indolent prime phase for an average term of 33 months (6–103) after the identification of the clone 5. Once the aggressive second phase achieved, the survival terms of the patients do not seem to significantly differ regardless of the clinical examination at the time of diagnosis. The exceptional case of a patient who lived for 18 years with such a peripheral clone without any substantial clinical expression was previously described but remains to this day an exception to our knowledge 6.

In our case, the main argument for the research of a T‐PLL clone was the typical morphology of lymphocytes on the blood smear, with a few distinctive cytological features (expansion of the cytoplasm, well‐mature chromatin with a frequent sole nucleolus) 7. The analysis of this case also shows the predominant role of the careful cytological analysis of the blood smear, even for a slight lymphocytosis. The early diagnosis also allows the implementation of regular hematological follow‐ups in order to be able to introduce the appropriate chemotherapy such as pentostatin or alemtuzumab 8. Currently, our patient is being followed every 2 months, and 15 months after the diagnosis, the clinical state remains unchanged and his cell count revealed a slowly increasing rate of lymphocytosis reaching 12.6 G/L with no other detectable abnormalities.

## Authorship

CJ: performed the cytologic and phenotypic analysis for diagnosis, synthesized the medical record, and wrote the paper. JF: supervised the laboratory steps for cytological diagnosis and follow‐up. CB: performed cytogenetics analyses and provided references and wrote the paper. EL: performed the clinical care of the patient in the in‐and outpatient setting. AB‐G: provided the scientific and bibliographic features. AGT: provided the scientific and bibliographic features and wrote the paper. CA: supervised the cytological analyses, synthesized the entire clinical history and wrote the paper.

## Conflict of Interest

None declared.
